# Apelin Affects the Progression of Osteoarthritis by Regulating VEGF-Dependent Angiogenesis and miR-150-5p Expression in Human Synovial Fibroblasts

**DOI:** 10.3390/cells9030594

**Published:** 2020-03-02

**Authors:** Yu-Han Wang, Shu-Jui Kuo, Shan-Chi Liu, Shih-Wei Wang, Chun-Hao Tsai, Yi-Chin Fong, Chih-Hsin Tang

**Affiliations:** 1 Graduate Institute of Biomedical Science, China Medical University, Taichung 40402, Taiwan; laecy0313@gmail.com; 2 School of Medicine, China Medical University, Taichung 40402, Taiwan; b90401073@gmail.com; 3 Department of Orthopedic Surgery, China Medical University Hospital, Taichung 40402, Taiwan; ritsai8615@gmail.com (C.-H.T.); yichin.fong@gmail.com (Y.-C.F.); 4 Department of Medical Education and Research, China Medical University Beigang Hospital, Yunlin 651, Taiwan; sdsaw.tw@yahoo.com.tw; 5 Department of Medicine, Mackay Medical College, New Taipei City 252, Taiwan; shihwei@mmc.edu.tw; 6 Department of Sports Medicine, College of Health Care, China Medical University, Taichung 40402, Taiwan; 7 Chinese Medicine Research Center, China Medical University, Taichung 40402, Taiwan; 8 Department of Biotechnology, College of Health Science, Asia University, Taichung 40402, Taiwan

**Keywords:** osteoarthritis, apelin, VEGF, synovial fibroblast, angiogenesis

## Abstract

Synovium-induced angiogenesis is central to osteoarthritis (OA) pathogenesis and thus a promising therapeutic target. The adipokine apelin (APLN) is involved in both OA pathogenesis and angiogenesis. We examined the role of APLN in synovium-induced angiogenesis by investigating the crosstalk between APLN and vascular endothelial growth factor (VEGF) expression in human OA synovial fibroblasts (OASFs). We found higher levels of APLN and VEGF expression in OA samples compared with normal samples. APLN-induced stimulation of VEGF expression and VEGF-dependent angiogenesis in OASFs was mitigated by FAK/Src/Akt signaling. APLN also inhibited levels of microRNA-150-5p (miR-150-5p), which represses VEGF production and angiogenesis. Analyses of an OA animal model showed that shAPLN transfection of OASFs rescued pathologic changes in OA cartilage and histology. Here, we found APLN enhances VEGF expression and angiogenesis via FAK/Src/Akt cascade and via downstream suppression of miR-150-5p expression. These findings help to clarify the pathogenesis of adipokine-induced angiogenesis in OA synovium.

## 1. Introduction

Osteoarthritis (OA) is associated with synovial inflammation, cartilage degradation, joint pain and swelling [1,2,3]. The synthesis of chondrolytic enzymes and proinflammatory mediators by inflamed OA synovium degrades cartilage, which aggravates synovial inflammation, creating a vicious cycle that is consolidated by synovial angiogenesis [4,5,6]. Synovium-induced angiogenesis is therefore a promising therapeutic target for OA [4,5].

Recognized risk factors for OA include age, gender, trauma, and obesity [7]. The fact that obesity is modifiable underscores its importance in the pathogenesis of OA [8]. An intuitive link between obesity and OA could be attributed to cartilage bearing an excess mechanical load. However, obesity-related adipokines could also disrupt the joint milieu through biologic signaling, including adipokine-induced angiogenesis [9]. An association between obesity, adipokines, adipokine-induced angiogenesis and OA would be crucial in the pathogenesis of OA, but little is known about any such association.

The endogenous ligand of APJ (apelin receptor), apelin (APLN), is expressed in various human tissues and cells, particularly vascular endothelial cells and adipose tissue [5,10]. Evidence of links between APLN/APJ and oxidative stress include the suppression by APLN/APJ of adipocyte-induced production and release of reactive oxygen species (ROS) and prevention of oxidative stress-linked cardiac hypertrophy; conversely, an active fragment of APLN can facilitate ROS generation in vascular smooth muscle cells, while APLN promotes the formation of atherosclerosis [9]. Moreover, synovial fluid APLN levels are increased in obese compared with nonobese individuals [11], and APLN upregulates the expression of catabolic factors metalloproteinase (MMP)-1, -3 and -9, as well as levels of the proinflammatory cytokine, interleukin 1 beta (IL-1β) [12], and plays a role in angiogenesis by facilitating the formation of a functional vascular network [13]. Furthermore, synovial fluid APLN concentrations are significantly and positively correlated with disease severity in patients with OA, while higher APLN and APJ transcript levels are found in OA cartilage compared with cartilage from healthy donors [14].

Vascular endothelial growth factor (VEGF) enhances endothelial cell migration, proliferation, and survival [15]. Significantly higher levels of VEGF expression are found in patients with OA compared with healthy controls [16], and variations in the *VEGF* gene significantly increase the susceptibility for developing OA [17], while high VEGF levels in plasma and synovial fluid are significantly and positively correlated with radiographic severity of knee OA [18]. Noncoding, single-stranded micro-ribonucleic acids (miRNAs) mediate gene expression at the post-transcriptional level and after base pairing with the seed sequence of target mRNA molecules, the 3′-untranslated region (3′-UTR), miRNAs can inhibit target gene expression [19]. OA pathogenesis involves various miRNAs [20]. A comprehensive review involving 46 bioinformatics studies that explored the correlation between microRNA expression and OA reported that chondrocyte autophagy was suppressed by miR-21 and enhanced by miR-146a, while chondrocyte apoptosis was suppressed by miR-9 and miR-24 and enhanced by miR-139 and miR-146a [21]. Other mechanisms that are also involved in interactions between microRNAs and OA include inflammation, chondrocyte homeostasis and differentiation and chondrocyte metabolic activities that alter extracellular matrix (ECM) production [22].

Considering the importance of synovium-induced angiogenesis in OA pathogenesis, we sought to elucidate existing crosstalk between APLN and VEGF in human osteoarthritis synovial fibroblasts (OASFs) and OA animal models (anterior cruciate transection; ACLT) in order to clarify the association between obesity, adipokine, adipokine-induced angiogenesis and OA. We speculated that APLN upregulates VEGF-dependent angiogenesis by mediating miRNA expression in OASFs and we sought to clarify the disease process involving adipokine-induced angiogenesis in OA.

## 2. Materials and Methods

### 2.1. Materials

Antibodies against VEGF (SC-507), focal adhesion kinase (FAK; SC-932), Src (SC-5226), Akt (SC-5298), and CD34(SC-74499) were bought from Santa Cruz (Santa Cruz, CA, USA). Antibodies targeting p-FAK (3283S), p-Src (2101S), p-AKT (4060S), and CD133(64326s) were obtained from Cell Signaling Technology, Inc. (Beverly, MA, USA). Antibodies against CD31(ab28364) was purchased from Abcam (Cambridge, MA, USA). Small interfering RNAs (siRNAs) against FAK (L-003164-00), Src (L-003110-00) and Akt (L-003000-00-0005) and their respective controls were purchased from Dharmacon (Lafayette, CO, USA). We purchased VEGF shRNA plasmids from the National RNAi Core (Taipei, Taiwan). Inhibitors for FAK (869288-64-2) were from Calbiochem (San Diego, CA, USA). A VEGF ELISA kit (DY293B) was purchased from R&D Systems (Minneapolis, MN, USA) and APLN ELISA (KA1681) kit was purchased from Abnova (Taipei, Taiwan). Inhibitors for Src (P0042), Akt (A6730) and all the chemicals not mentioned above were supplied by Sigma-Aldrich (St. Louis, MO, USA).

### 2.2. Cell Culture

Human OASFs were harvested from synovial tissue taken from the suprapatellar pouch of the OA knees of 15 patients (5 males and 10 females; mean age 73.3 years) undergoing knee replacement surgery for end-stage knee OA. Written informed consent was obtained from all patients. After processing synovial tissue in 0.5 mL 1% type IV collagenase, the cells were cultured in DMEM medium containing 10% fetal bovine serum (FBS), 50 μg/mL streptomycin and 50 U/mL penicillin (Invitrogen; Carlsbad, CA, USA). Single-cell cultures from passages 3 to 6 were used in the experiments [1,23].

Human endothelial progenitor cells (EPCs) were prepared according to our previous protocols [24,25,26], after we had obtained approval from the Institutional Review Board (IRB) of Mackay Medical College, New Taipei City, Taiwan (reference number: P1000002). Peripheral blood was collected from healthy donors after they completed written informed consent forms. Mononuclear cells were isolated from blood components using centrifugation on Ficoll-Paque PLUS (Amersham Biosciences, Uppsala, Sweden). EPCs were characterized and maintained using methods described in our previous reports [27,28,29].

### 2.3. Clinical Samples

Serum and synovial tissue samples were obtained from patients with OA undergoing knee replacement surgery and also patients undergoing arthroscopy after trauma/joint derangement, who served as normal controls, in China Medical University Hospital, Taichung, Taiwan. All subjects gave their informed consent for inclusion before they participated in the study. The study was conducted in accordance with the Declaration of Helsinki, and the protocol was approved by the Ethics Committee of by the Institutional Review Board (IRB) of China Medical University Hospital (CMUH108-REC3-039) and all methods were performed in accordance with the hospital’s IRB guidelines and regulations.

### 2.4. RT-qPCR of mRNA and miRNA

A TRIzol kit (MDBio Inc., Taipei, Taiwan) extracted total RNA from human synovial fibroblasts, then 1 µg of total RNA was reverse transcribed into cDNA using oligo(dT) primers [30]. Real-time quantitative PCR (RT-qPCR) analysis was performed using Taqman^®^ One-Step RT-PCR Master Mix (Applied Biosystems, Foster City, CA, USA). Sequence-specific primers and Taqman^®^ probes were used to add 2 µL of cDNA template to each 25-μL reaction. The sequences for all target gene primers and probes were purchased commercially. Glyceraldehyde 3-phosphate dehydrogenase (GAPDH) was used as an endogenous control to normalize expression data (Applied Biosystems). RT-qPCR assays were carried out in triplicate in a StepOnePlus sequence detection system (Applied Biosystems). The cycling conditions were as follows: initial 10-min polymerase activation at 95 °C followed by 40 cycles at 95 °C for 15 s and 60 °C for 60 s. The threshold was set above the non-template control background and within the linear phase of the target gene amplification to calculate the cycle number at which the transcript was detected (denoted as CT).

For the miRNA assay, cDNA was synthesized from total RNA (100 ng) using the TaqMan MicroRNA Reverse Transcription Kit (Applied Biosystems). The reactions were incubated first at 16 °C for 30 min and then at 42 °C for 30 min followed by inactivation at 85 °C for 5 min. The reactions were then incubated in a 96-well plate at 50 °C for 2 min, 95 °C for 10 min, followed by 30 cycles of 95 °C for 15 s and 60 °C for 1 min using the StepOnePlus sequence detection system. Relative quantification of gene expression was performed using the endogenous control gene (U6). The threshold cycle (CT) was defined as the fractional cycle number at which the fluorescence passed the fixed threshold. Relative expression was calculated using the comparative CT method. The primer sequences used were defined as VEGF-A forward primer: (GCAGAATCATCACGAAGTGG); reverse primer: (GCATGGTGATGTTGGACTCC); GAPDH forward primer: (ACCACAGTCCATGCCATCAC); and reverse primer: (TCCACCACCCTGTTGCTGTA).

### 2.5. Western Blot Analysis

Cell lysate was resolved by sodium dodecyl sulfate-polyacrylamide gel electrophoresis and transferred to Immobilon polyvinyl difluoride membranes (Immobilon P, Millipore). The blots were blocked with 4% Bovine serum albumin (BSA) for 1 h at room temperature and then probed with rabbit anti-human antibodies against primary antibody (1:1000) for 1 h at room temperature. After 3 washes, the blots were subsequently incubated with donkey anti-rabbit peroxidase-conjugated secondary antibody (1:3000) for 1 h at room temperature. Enhanced chemiluminescent imaging of the blots was visualized using the UVP Biospectrum system (UVP, Upland, CA, USA) [31,32].

### 2.6. ELISA Assay

OASFs were cultured in 24-well plates until they reached 90% confluence before being changed to serum-free medium, in which they were treated with APLN for 24 h with or without the transfection of siRNAs or inhibitors. The conditioned medium (CM) was collected and VEGF levels were quantified with the VEGF ELISA kit.

Serum from patients with OA or normal controls were collected. VEGF levels were quantified using the VEGF ELISA kit, and APLN levels were quantified using the APLN ELISA kit.

### 2.7. Analysis of the Gene Expression Omnibus (GEO) Dataset

We retrieved data from the GEO database records (accession code: GSE46750), which compares non-inflammatory areas to inflammatory areas of the synovium from 12 patients with osteoarthritis. The HotPlot was drawn using TreeView software v3.0 (http://jtreeview.sourceforge.net/).

### 2.8. Transient Transfection

Human synovial fibroblasts were cultured in a 6-well plate and miR-150-5p mimic or APLN shRNA were transfected into the cells using Lipofectamine™ 2000 (Invitrogen; Carlsbad, CA, USA).

ON-TARGET*plus* siRNAs (100 nM) was transiently transfected with DharmaFECT1 transfection reagent (Lafayette, CO, USA), according to the manufacturer’s instructions.

### 2.9. Plasmid Construction and Luciferase Assays

The wild-type VEGF-3′-UTRs fragments (specific for miR-150-5p) were cloned into the pmirGLO-luciferase reporter vector. The primer sequences used were defined as VEGF forward primer: (CGGCTAGCGACACACCCACCCACATACA); the reverse primer: (GGCTCGAGTCTCCTCCTCTTCCCTGTCA). The mutant 3′UTR region of VEGF mRNA (mt-IL-VEGF-3′-UTR) was purchased from Invitrogen. The Dual-Luciferase^®^ Reporter Assay System was bought from Promega (Madison, WI, USA). Luciferase activity was assayed using the method described in our previous publications [33,34].

### 2.10. Tube Formation Assay

Matrigel (BD Biosciences, Bedford, MA, USA) coated on 48-well plates and EPCs (2 × 10^4^ per 100 μL) were resuspended in MV2 serum-free medium with the indicated APLN concentration and then added to the wells. After 6 h of incubation at 37 °C, EPC tube formation was assessed with a photomicroscope, and each well was photographed at 200× magnification. The number of tube branches was calculated using MacBiophotonics ImageJ software (v1.51, National Institutes of Health, Bethesda, MD, USA) [15,35].

### 2.11. Transwell Migration Assay

EPC cell migration assays were used with Transwell inserts (Corning/Costar, Corning, NY, USA) in 24-well plates, and 2 × 10^4^ EPCs were applied to the upper chamber with 10% FBS MV2 medium and the culture medium combined with 20% FBS MV2 complete medium in lower chamber. After incubating the plates for 16 h at 37 °C in 5% CO_2_, the cells were fixed in 3.7% formaldehyde solution for 15 min and stained with 0.05% crystal violet in phosphate buffered saline (PBS) for 15 min. Cells on the upper side of the filters were removed with cotton-tipped swabs, and the filters were washed with PBS. Cells on the underside of the filters were examined and counted under a microscope [15,36].

### 2.12. Chick Chorioallantoic Membrane Assay

Fertilized chicken eggs were used in chorioallantoic membrane (CAM) assay. In vivo angiogenic activity was assessed using the CAM assay, as described previously [15,37].

### 2.13. In Vivo Matrigel Plug Assay

Four-week-old male nude mice were subcutaneously injected with 0.15 mL of Matrigel containing the indicated OASFs CM. On day 7, the Matrigel plugs were harvested; the methods used to measure hemoglobin concentrations were described previously [15,37,38].

### 2.14. Experimental OA Model

Male Sprague-Dawley (SD) rats (8 weeks of age, weighing 300–350 g) were procured from the National Laboratory Animal Center in Taiwan and maintained under conditions complying with the Guidelines of the Animal Care Committee of China Medical University, Taichung, Taiwan. We followed the protocol established by Wang et al. for our rat model of anterior cruciate transection (ACLT) to induce OA [39]. In brief, the left knee was prepared in a surgically sterile fashion. The ACL fibers were transected with a scalpel and the entire medial meniscus was excised using a medial parapatellar mini-arthrotomy. The joint surface was washed with sterile saline solution, and both capsule and skin were sutured after ACL transection and medial meniscectomy. Ampicillin 50 mg/kg body weight was administered for 5 days after the surgery. After surgery (day 0), the rats were divided into 3 groups (*n* = 8 per group): a control group, an ACLT group, and an shAPLN-transfected ACLT group. For 6 weeks, the shAPLN-transfected ACLT group was given weekly intra-articular injections of ~7.1 × 10^6^ plaque-forming units (PFU) of shAPLN. All rats were allowed to move freely in plastic cages until necropsy at 10 weeks post-surgery. All animal procedures were approved and performed in accordance with the guidelines of the Institutional Animal Care and Use Committee of China Medical University (CMUIACUC-2019-134).

### 2.15. Micro-Computed Tomography (Micro-CT) Imaging

The micro-computed tomography (micro-CT) assessment protocol was based upon our previous publications [15,38]. Rat knee joints were extracted promptly after sacrifice and fixed in 3.7% formaldehyde for micro-CT imaging. Three-dimensional microstructural volumes from micro-CT scans were analyzed using Skyscan software v1.18 (CTAn/Bruker, Cambridge, UK) [15].

### 2.16. Histological Analysis

Immunohistochemistry (IHC) staining was performed on serial sections of the extracted rat knee joints. Joint sections were deparaffinized with xylene and rehydrated with ethanol. The specimens were stained with specific primary antibodies according our previous protocol [40]. Some samples were also stained with Safranin O-fast Green [24,38]. The OARSI scoring system proposed by Glasson et al. was used to assess cartilage injury in the tibiofemoral joints in a blinded fashion [41].

### 2.17. Statistics

All statistical analyses were carried out using GraphPad Prism v5.0 (GraphPad Software Inc., San Diego, CA, USA), and all values are expressed as the mean ± S.D. Differences between selected pairs from the experimental groups were analyzed for statistical significance using the paired sample *t*-test for in vitro analyses and 1-way ANOVA followed by Bonferroni testing for in vivo analyses. The statistical difference was considered to be significant if the *p*-value was <0.05.

## 3. Results

### 3.1. Apelin Expression is Positively Associated with VEGF Expression Among the OA Patients

**Figure 1:**
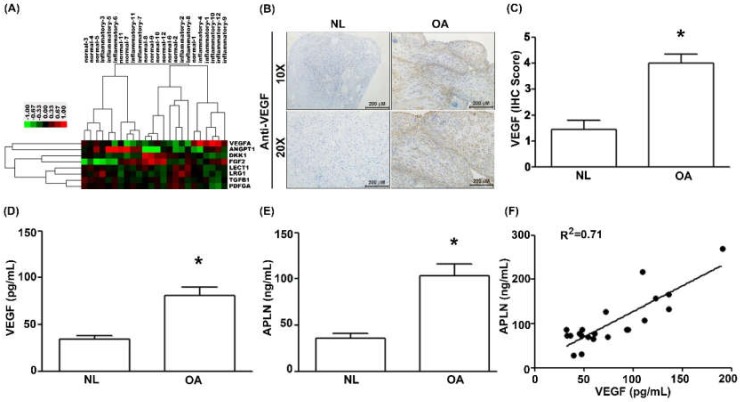
The expression of apelin (APLN) is positively correlated with the expression of vascular endothelial growth factor (VEGF) among osteoarthritis (OA) patients. (**A**) Expression levels of APLN were retrieved from the Gene Expression Omnibus (GEO) database records (accession code: GSE46750), which compares non-inflammatory areas to inflammatory areas of the synovium from 12 patients with OA. (**B**,**C**) IHC staining revealed higher levels of VEGF expression in OA synovial tissues (*n* = 8) compared with non-OA tissues (*n* = 5). (**D**) The ELISA assay revealed higher levels of serum VEGF expression among OA patients (*n* = 21) compared with controls (*n* = 21) and also (**E**) higher levels of serum APLN expression among OA patients compared with controls. (**F**) A positive correlation between VEGF and APLN expression in serum OA samples. NL = normal controls. * *p* < 0.05 compared with normal group.

To decipher crosstalk between APLN and VEGF in the OA cohort, we examined the GEO database records, which demonstrated higher levels of VEGF expression in inflamed tissue compared with normal tissue in OA patients (Figure 1A). Under IHC staining, substantially higher VEGF expression was found in OA tissue (Figure 1B,C), and Supplementary Materials Figure S1). Serum concentrations of VEGF (Figure 1D; *p* = 0.025) and APLN (Figure 1E; *p* = 0.019) were significantly higher in OA patients, and a positive significant correlation was observed between serum VEGF and APLN concentrations (Figure 1F; Spearman R = 0.7154; *p* = 0.003).

### 3.2. APLN Stimulates VEGF Expression and Facilitates Angiogenesis

**Figure 2:**
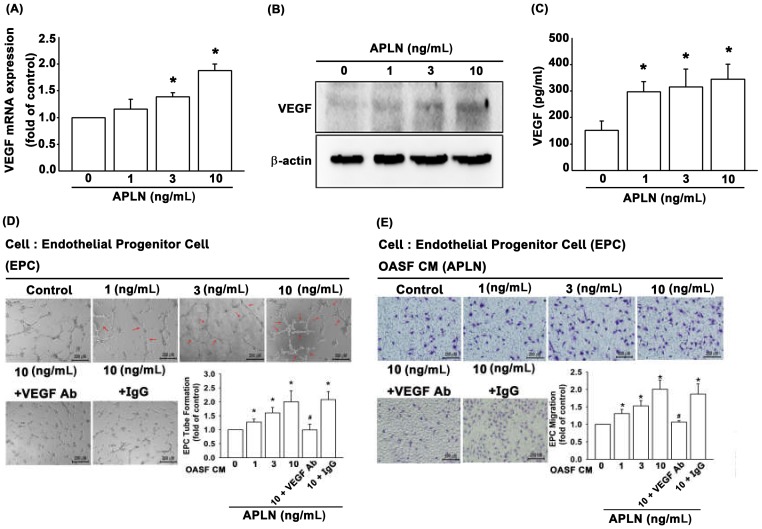
APLN stimulates VEGF expression and angiogenesis capacity in osteoarthritis synovial fibroblasts (OASFs). (**A**) APLN (0, 1, 3, or 10 ng/mL) was administered to OASFs for 24 h, and the extent of VEGF transcription was examined using real-time quantitative PCR (RT-qPCR) (*n* = 4). (**B**) OASFs were incubated under various APLN concentrations for 24 h, and VEGF translation levels were examined using Western blot (*n* = 3). (**C**) OASFs were cultured with APLN for 24 h, and excreted VEGF protein levels were assayed using ELISA assay (*n* = 5). The culture medium was subsequently collected, and culture medium from OASFs incubated with APLN was further treated with VEGF antibody and IgG. All culture medium (with and without VEGF/IgG antibody treatment) were subjected to endothelial progenitor cell (EPC) tube formation (*n* = 5) (**D**) and EPC migration (**E**) assays (*n* = 5). * *p* < 0.05 compared with control; # *p* < 0.05 compared with the APLN-treated group.

It is unclear as to whether any crosstalk exists between APLN and VEGF in synovium-induced angiogenesis. We found that APLN (0–10 ng/mL) dose-dependently stimulated transcription (SD values of 1.0, 1.14 ± 0.133, 1.358 ± 0.075, 1.881 ± 0.119, respectively) and translation (Figure 2A,B) of VEGF. APLN stimulated the excretion of VEGF protein by OASFs in a concentration-dependent manner (Figure 2C). The effects of APLN-mediated angiogenesis in OASFs were evaluated using EPC tube formation and migration assays [24]. CM from APLN-treated OASFs enhanced tube formation (Figure 2D) and migration (Figure 2E) in EPCs. EPC tube formation and migration induced by CM obtained from APLN-treated OASFs was abolished by VEGF antibody, whereas immunoglobulin G (IgG) had no such effects (Figure 2D,E), suggesting that APLN induces angiogenesis in a VEGF-dependent manner.

### 3.3. APLN Stimulates VEGF Expression via the Phosphorylation of FAK/Src/Akt

**Figure 3:**
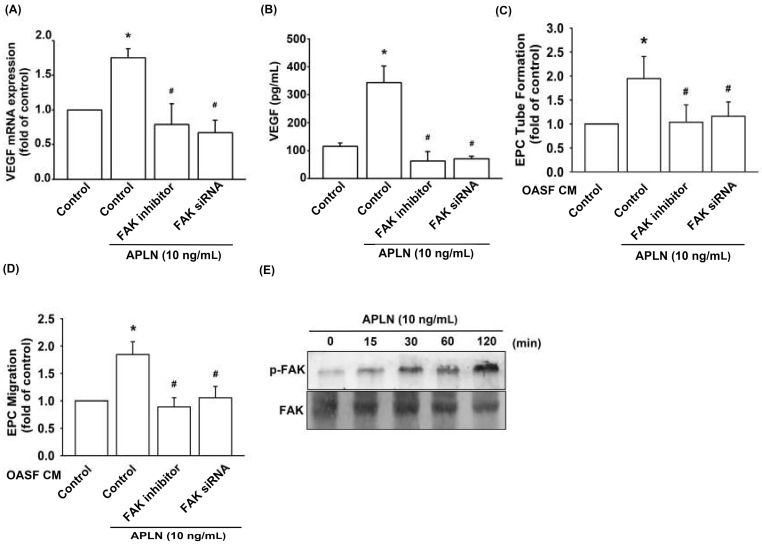
Focal adhesion kinase (FAK) phosphorylation is involved in APLN-induced VEGF synthesis. (**A**,**B**) OASFs were pretreated with a FAK inhibitor for 30 min or FAK small interfering RNAs (siRNA) for 24 h, then incubated with APLN (10 ng/mL) for 24 h. (**A**) VEGF levels were examined using RT-qPCR (*n* = 4) and ELISA assay (*n* = 5). (**C**,**D**) OASF culture medium collected after APLN incubation was subjected to EPC (**C**) tube formation (*n* = 5) and (**D**) migration assays (*n* = 5). (**E**) OASFs were incubated with APLN for the indicated time intervals, and the extent of FAK phosphorylation was examined using Western blot analysis. * *p* < 0.05 compared with control; # *p* < 0.05 compared with the APLN-treated group.

To determine the role of FAK in APLN-enhanced VEGF production, we pretreated OASFs with a FAK inhibitor or transfected them with a FAK siRNA before APLN administration. The RT-qPCR (Figure 3A) and ELISA assays (Figure 3B) confirmed significant amelioration of APLN-enhanced VEGF expression in OASFs when pretreated with a FAK inhibitor or FAK siRNA. Pretreating OASFs with a FAK inhibitor and FAK siRNA before APLN administration also mitigated EPC tube formation (Figure 3C) and migration (Figure 3D) induced by APLN-treated OASF CM. APLN led to a time-dependent increase in the phosphorylation of FAK (Figure 3E). These findings demonstrate that FAK activation is involved in APLN-stimulated VEGF expression and EPC angiogenesis.

**Figure 4:**
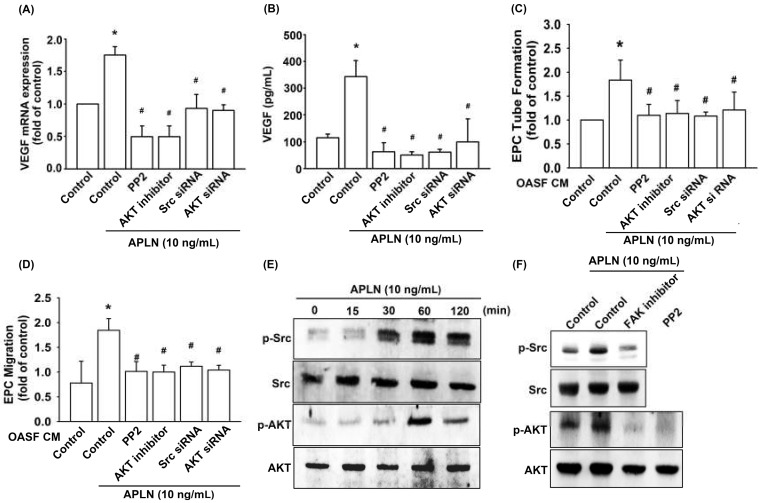
Src and Akt phosphorylation are involved in APLN-induced VEGF synthesis. (**A**,**B**) OASFs were pretreated with Src/Akt inhibitors for 30 min or Src/Akt siRNAs for 24 h, then incubated with APLN for 24 h. (**A**) VEGF mRNA levels were examined using RT-qPCR (*n* = 4), and (**B**) excreted VEGF protein levels were quantified using ELISA assay (*n* = 5). (**C**,**D**) After pretreatment with Src/Akt inhibitors or Src/Akt siRNA and incubation with APLN, the culture medium was collected and subjected to EPC (**C**) tube formation (*n* = 5) and (**D**) migration assays (*n* = 5). (**E**) OASFs were incubated with APLN, and the extent of Src/Akt phosphorylation was examined using Western blot analysis (*n* = 3). (**F**) OASFs pretreated with a FAK inhibitor before APLN incubation were assessed for the extent of Src/Akt phosphorylation, while OASFs pretreated with a Src inhibitor before APLN incubation were assessed for Akt phosphorylation (*n* = 3). * *p* < 0.05 compared with control; # *p* < 0.05 compared with the APLN-treated group.

Pretreatment with an Src-specific inhibitor (PP2) or transfection with Src siRNA diminished APLN CM-enhanced VEGF transcription (Figure 4A) and protein excretion (Figure 4B). The pretreatment of OASFs with Src inhibitor and Src siRNA before APLN administration mitigated EPC tube formation (Figure 4C) and EPC migration (Figure 4D) stimulated by-APLN-treated OASF CM. APLN led to a time-dependent increase in Src phosphorylation (Figure 4E).

Pretreatment with an Akt inhibitor or transfection with Akt siRNA diminished APLN CM-enhanced VEGF transcription (Figure 4A) and protein excretion (Figure 4B). Pretreatment of OASFs with an Akt inhibitor and Akt siRNA before APLN administration mitigated EPC tube formation (Figure 4C) and migration (Figure 4D) stimulated by ApPLN-APLN treated OASF CM. APLN led to a time-dependent increase in the Akt phosphorylation (Figure 4E). APLN-induced Src phosphorylation was mitigated by a FAK inhibitor, and APLN-induced Akt phosphorylation was ameliorated by a FAK inhibitor and Src inhibitor (Figure 4F). These findings suggest that APLN enhances VEGF expression via the serial phosphorylation of FAK/Src/Akt (upstream to downstream) cascade.

### 3.4. APLN Enhances VEGF Expression and Angiogenesis by Inhibiting miR-150-5p Synthesis

**Figure 5:**
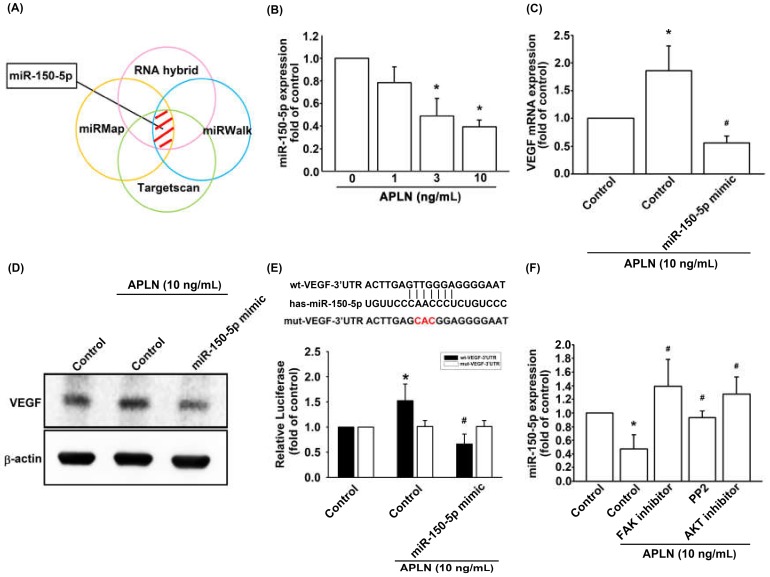
Apelin suppression of miR-150-5p enhances VEGF production. (**A**) Open-source software (TargetScan, miRMap, RNAhybrid, and miRWalk) was employed to determine which miRNAs could potentially inhibit VEGF transcription. (**B**) OASFs were incubated with APLN. miR-150-5p expression levels were examined using RT-qPCR assay (*n* = 4). (**C**,**D**) OASFs were transfected with miR-150-5p mimic and then stimulated by APLN. (**C**) VEGF transcription and (**D**) translation levels were examined using RT-qPCR (*n* = 4) and ELISA assays (*n* = 5). (**E**) OASFs were transfected with the mt-VEGF-3′UTR plasmid with or without miRNA-150-5p mimic, then incubated under APLN. Relative luciferase activity reflected VEGF promoter activity (*n* = 6). (**F**) OASFs were treated with FAK, Src, or Akt inhibitor then incubated with APLN. miR-150-5p expression levels were examined using RT-qPCR assay (*n* = 4). * *p* < 0.05 compared with control; # *p* < 0.05 compared with the APLN-treated group.

The intricate networks of miRNAs in synovium-induced angiogenesis are little understood. We employed open-source software (TargetScan; http://www.targetscan.org/vert_72/, miRMap; https://mirmap.ezlab.org/, RNAhybrid; http://alk.ibms.sinica.edu.tw/cgi-bin/RNAhybrid/RNAhybrid.cgi, and miRWalk; http://zmf.umm.uni-heidelberg.de/apps/zmf/mirwalk2/) to identify candidate miRNAs that could potentially inhibit VEGF transcription (Figure 5A). Among the 15 screened miRNAs that could potentially bind to the 3′UTR region of VEGF mRNA, the expression of miR-150-5p was most substantially inhibited by APLN stimulation. To validate these findings, we compared the expression levels of miR-150-5p in OASFs under graded APLN stimulation. We found that APLN (1–10 ng/mL) suppressed the extent of miR-150-5p expression in a concentration-dependent manner (Figure 5B). To define whether APLN enhances VEGF expression by suppressing miR-150-5p synthesis, we transfected the OASFs with miR-150-5p, which reversed APLN-enhanced VEGF transcription and translation (Figure 5C,D). We also used the luciferase reporter vector, including the wild-type 3′UTR of VEGF mRNA (wt-VEGF-3′UTR) and the vector containing mismatches in the miR-150-5p binding site (mt-VEGF-3′UTR), to define whether miR-150-5p disrupts VEGF transcription (Figure 5E). We found that miR-150-5p mimic mitigated APLN-induced luciferase activity in the wt-VEGF-3′UTR plasmid, but not in the mt-VEGF-3′UTR plasmid (Figure 5E). In addition, the FAK, Src, and Akt inhibitors reversed APLN-inhibited miR-150-5p expression (Figure 5F). miR-150-5p appears to disturb VEGF transcription via binding to the 3′UTR region of VEGF mRNA, and miR-150-5p expression is suppressed by FAK/Src/Akt phosphorylation induced by upstream APLN stimulation.

### 3.5. Infection of shAPLN Reduces VEGF-Mediated Angiogenesis

**Figure 6:**
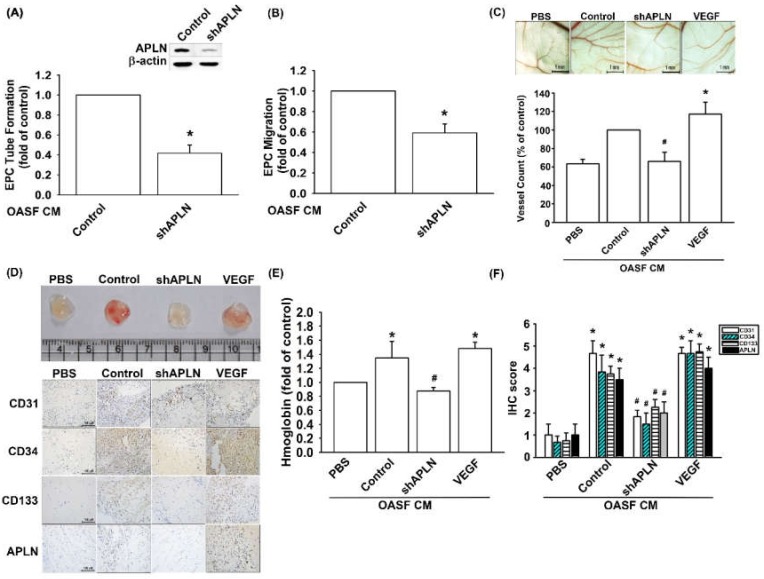
APLN knockdown in OASFs decreases VEGF-dependent angiogenesis. (**A**,**B**) Culture medium from control-shRNA and shAPLN-transfected OASFs was applied to EPCs for 24 h; EPC capillary-like structure formation and cell migration was examined using tube formation (*n* = 5) and migration assays (*n* = 5). (**C**) The harvested culture medium was applied to 6-day-old fertilized chick embryos for 4 days. Chorioallantoic membranes (CAMs) were then examined using microscopy and photographed, and vessels counted (*n* = 3). (**D**) Matrigel plugs containing the harvested culture medium were subcutaneously injected into the flanks of nude mice (*n* = 6). (**E**) After 7 days, the plugs were photographed, and hemoglobin levels were quantified. (**D**) Plug specimens were immunostained with antibodies against CD31, CD34, CD133, and APLN and (**F**) quantified using the IHC score. * *p* < 0.05 compared with control; # *p* < 0.05 compared with the APLN-treated group.

To confirm APLN-mediated VEGF-dependent angiogenesis in vivo, shAPLN was infected into OASFs 1 and 7 days before incubation with APLN. CM after APLN incubation was collected for analysis. shAPLN abolished CM-enhanced EPC tube formation and migration (Figure 6A,B). The collected CM was later employed for the CAM and Matrigel plug assays, while VEGF (50 ng/mL) served as the positive control. CM collected after APLN incubation demonstrated similar angiogenesis capacity to that of the VEGF positive control. However, APLN knockdown by shAPLN transfection lowered the vessel count under CAM assay (Figure 6C) and mitigated microvessel formation in Matrigel plugs, as confirmed by hemoglobin levels and CD31, CD34, CD133, and APLN IHC staining (Figure 6D–F, and Supplementary Figure S2).

### 3.6. Infection of shAPLN Mitigates in Vivo Severity of OA

**Figure 7:**
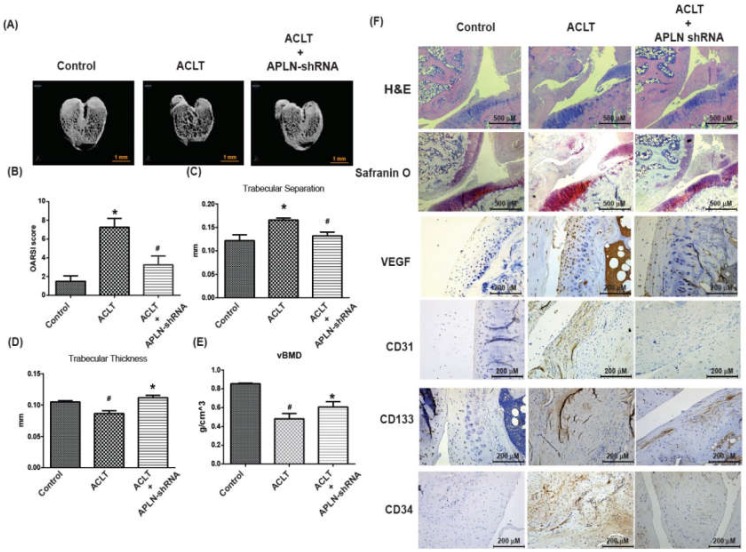
shAPLN ameliorates histologic severity of OA. (**A**) Micro-CT images of the control knee, anterior cruciate transection (ACLT) knee, and shAPLN-transfected ACLT knee (*n* = 8). (**B–E**) OARSI scores (**B**), micro-CT parameters, including trabecular separation (**C**), trabecular thickness (**D**), and volumetric bone mineral density (**E**), of the control knee, ACLT knee, and shAPLN-transfected ACLT knee. (**F**) Specimens from the control knee, ACLT knee, and shAPLN-transfected ACLT knee were immunostained with Safranin-O, VEGF, CD31, CD133, and CD34. * *p* < 0.05 compared with control; # *p* < 0.05 compared with the APLN-treated group.

**Figure 8:**
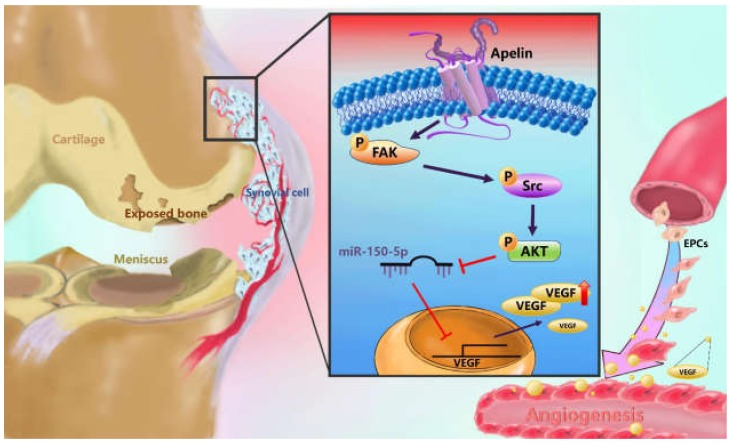
Schematic diagram summarizes the mechanism whereby Apelin upregulates VEGF expression in human synovial fibroblasts. APLN induces VEGF expression and angiogenesis through the FAK/Src/Akt signaling cascade and reduces miR-150-5p expression in OASFs. FAK: focal adhesion kinase; VEGF: vascular endothelial growth factor; EPCs: Human endothelial progenitor cells.

To validate the in vivo role of APLN, we investigated the effects of shRNA-mediated APLN knockdown on OA severity in our ACLT OA model. The ACLT OA knees and control knees were injected with ~7.1 × 10^6^ PFU shAPLN once weekly for 4 weeks, and the rats were later sacrificed. The knee joints injected with shAPLN were subsequently extracted for histologic study. Under micro-CT imaging, shAPLN administration restored the integrity of the subchondral bone architecture in the ACLT OA model (Figure 7A). In comparison to control samples, subchondral bone from rats with ACLT-induced OA had significantly higher trabecular separation (TS) values (Figure 7C), significantly lower trabecular thickness (TT) values (Figure 7D), and significantly lower bone mineral density (vBMD) values (Figure 7E). All of these ACLT-induced effects were reversed by shAPLN transfection. Compared with the control samples, those from the ACLT group demonstrated significantly higher OARSI scores (Figure 7B), significantly lower cartilage thickness under Safranin-O staining, and significantly higher expression of VEGF, CD31, CD133, and CD34. ACLT-induced histologic changes were reversed by shAPLN transfection (Figure 7F and Supplementary Figure S3). Figure 8 represents the overall study results.

## 4. Discussion

APLN plays an integral role in the obesity/adipokine/adipokine-induced angiogenesis–OA network [9,13,14]. APLN increases transcript levels of MMP-1, -3 and -9, and protein synthesis of IL-1β in chondrocytes, and leads to substantial degradation of glycoprotein [12]. APLN also dose-dependently increases angiogenesis responses, including endothelial cell migration, proliferation, and the capillary tube-like structure formation of endothelial cells [42]. Tissue hypoxia increases APLN transcription and neovascularization [13]. Up until now, however, the impact of APLN on synovium-induced angiogenesis has not been fully delineated. In this study, we found that APLN enhances the expression of VEGF and VEGF-dependent angiogenesis in OASFs by stimulating FAK, Src, and Akt phosphorylation and inhibiting the downstream expression of miR-150-5p.

Many miRNAs are involved in OA pathogenesis [43,44]. Researchers have reported that downregulating miR-150 alleviates inflammatory injury induced by IL-1 due to overexpression of Kruppel-like factor 2 [45]. However, little is known about the impact of miR-150 on OASFs. In our study, we used open-source software (TargetScan, miRMap, RNAhybrid, and miRWalk) to predict which miRNAs potentially interfere with APLN transcription. Our analysis of candidate miRNAs that can potentially interfere with the transcription of VEGF identified that miR-150-5p was inhibited to the greatest extent by VEGF. We also found that transfecting OASFs with miR-150-5p mimics ameliorated APLN-enhanced VEGF expression. These results highlight the importance of miR-150-5p in the synovium-induced angiogenesis.

FAK, Src, and Akt are involved in the processes of angiogenesis and pathogenesis of OA. Treatment of osteochondral explants with FAK and Src inhibitors prior to a death-inducing impact load decreased chondrocyte death at 24 h post-impact [46]. Researchers have also found that activation of the Akt signaling pathway promotes chondrocyte anabolism, while impaired activity of Akt signaling suppresses chondrocyte anabolism during the development of OA [47]. The aforementioned studies demonstrate the role of FAK, Src, and Akt on chondrocyte metabolism. However, the impact of these molecules on synovium-induced angiogenesis is not clear. In this study, we demonstrate that APLN stimulates VEGF expression via FAK, Src, and Akt phosphorylation, which underlines the importance of FAK, Src, and Akt in synovium-induced angiogenesis. Transcriptional and post-transcriptional modulations are known to affect miRNA activation and inhibition [19]. In our study, administration of FAK, Src, and Akt inhibitors mitigated APLN-inhibited miR-150-5p expression in OASFs, indicating that APLN suppresses the expression of miR-150-5p via the FAK/Src/Akt signaling cascade.

The expression of APLN and its receptor APJ comprises a system that acts as a critical regulator of various physiological functions, such as glycometabolism, liver disease, and macrophage activation [48,49,50]. A limitation of our study is that we did not examine APJ expression in synovial tissues. It remains to be determined as to whether similar interactions are involved in APLN-induced VEGF-mediated angiogenesis in OA synovial fibroblasts. Furthermore, our study has not clarified whether the proposed cascade modulates miR-150-5p expression via transcriptional or post-transcriptional modulation. This aspect deserves further investigation.

## 5. Conclusions

We demonstrate that APLN induces VEGF expression and angiogenesis through the FAK/Src/Akt signaling cascade and reduces miR-150-5p expression in OASFs. These results improve our understanding about the role of APLN in synovium-induced angiogenesis in OA. Mitigating APLN-induced angiogenesis might be a useful strategy in OA management, particularly in patients who are obese.

## Supplementary Materials

The following are available online at https://www.mdpi.com/2073-4409/9/3/594/s1, Figure S1. The large image of Figure 1B, Figure S2. The large image of Figure 6D, Figure S3. The large image of Figure 7F.
