# Supplementary material for: Apelin Affects the Progression of Osteoarthritis by Regulating VEGF-Dependent Angiogenesis and miR-150-5p Expression in Human Synovial Fibroblasts
Source: Cells. 2020 Mar 2;9(3):594. doi: 10.3390/cells9030594 (PMC7140420; doi:10.3390/cells9030594)
Supplement: Supplementary file 1 [file cells-09-00594-s001.pdf]

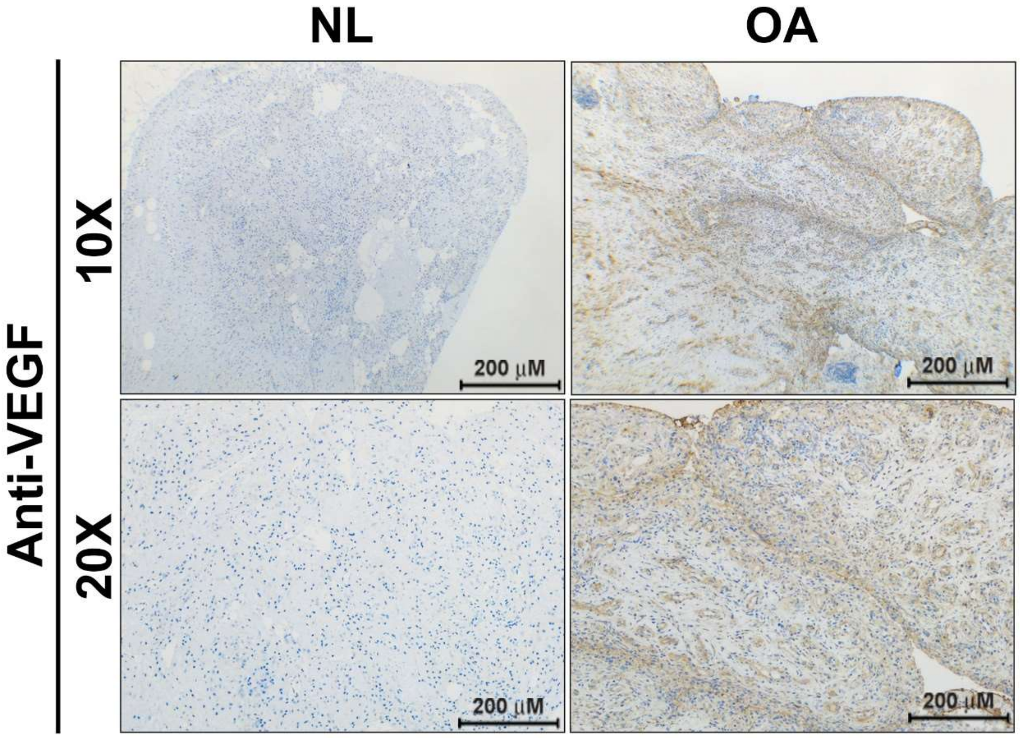

Figure 1. The large image of figure 1B.

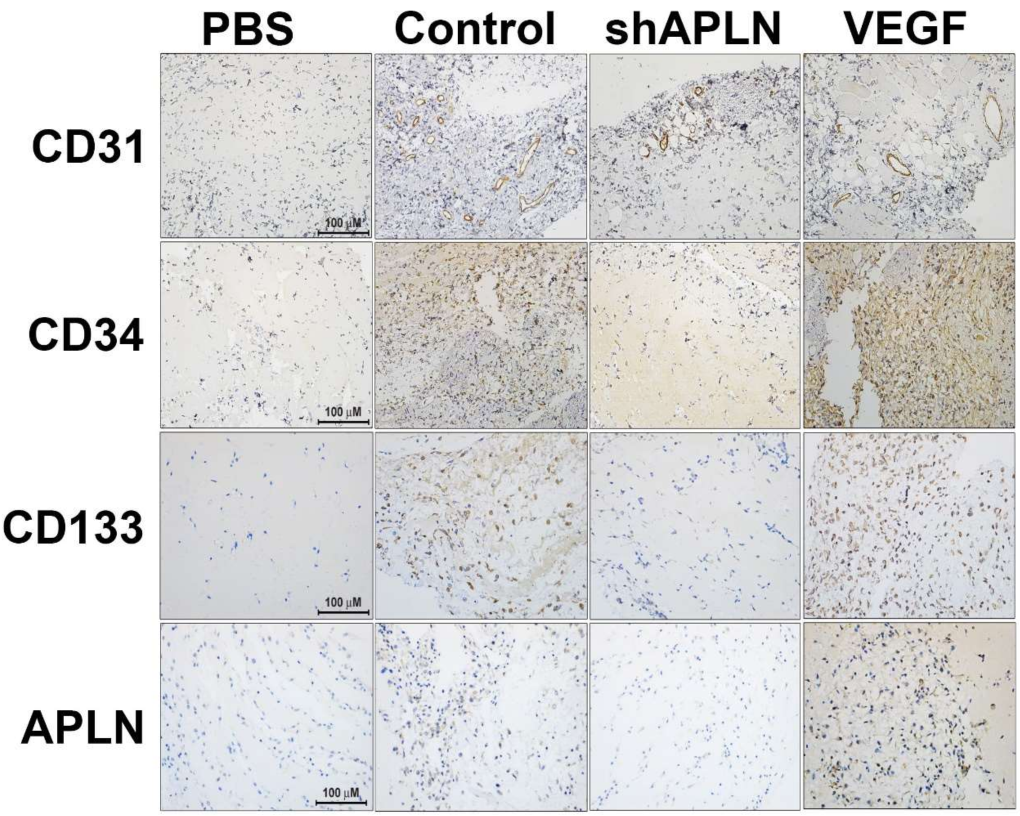

Figure 2. The large image of figure 6D.

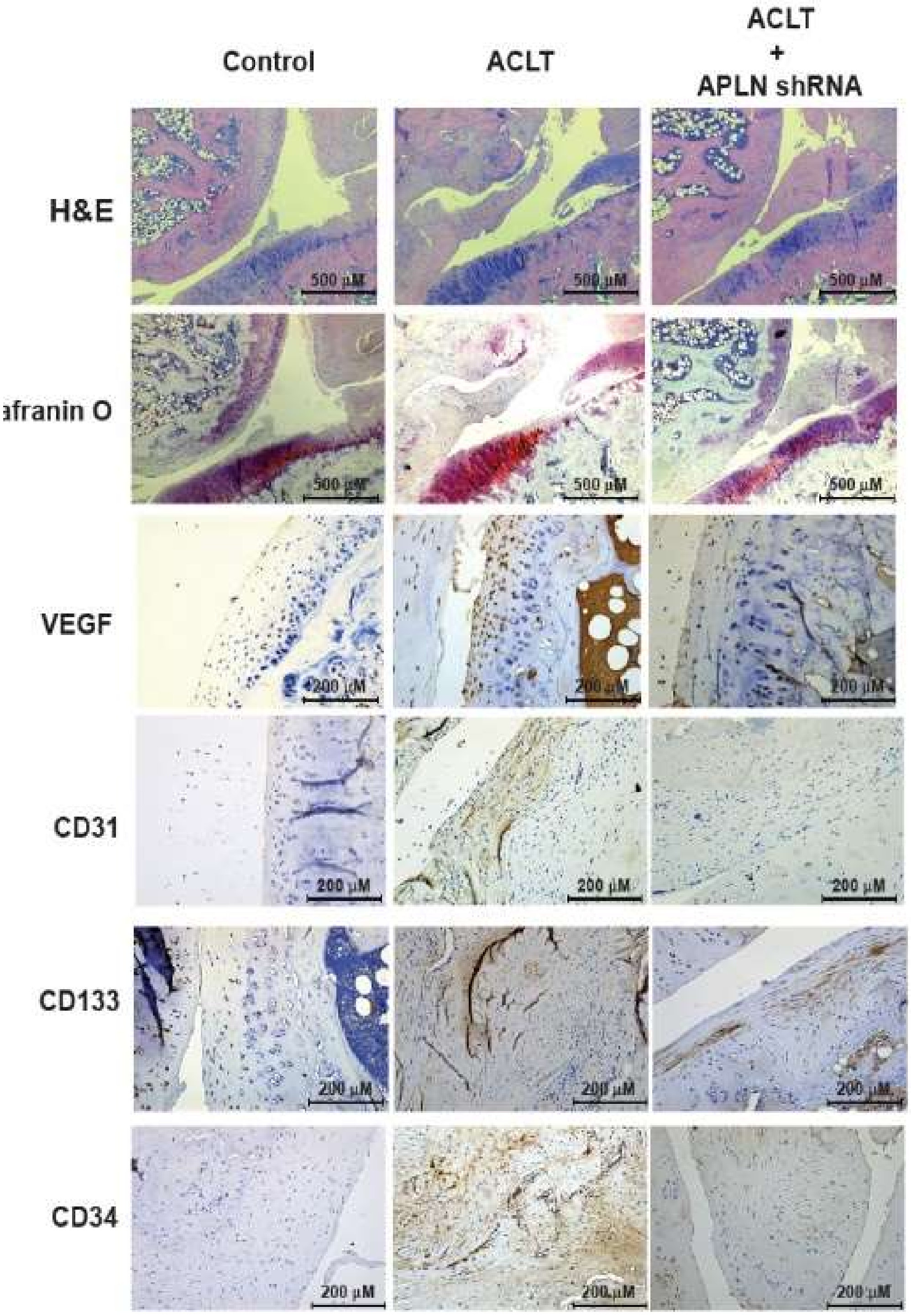

Figure 3. The large image of figure 7F.
